# Supplementary material for: Comparative multiomics analysis of cell physiological state after culture in a basket bioreactor
Source: Sci Rep. 2022 Nov 23;12:20161. doi: 10.1038/s41598-022-24687-4 (PMC9686226; doi:10.1038/s41598-022-24687-4)
Supplement: Supplementary file 1 — Supplementary Information 1. [file 41598_2022_24687_MOESM1_ESM.zip › raw data/Transcriptomic raw data/6.Enrichment/1.GO/TE1vsTC1/GSEA/TE1/TRANSLATIONAL_INITIATION/TRANSLATIONAL_INITIATION(GO_0006413).html]

Details for gene set TRANSLATIONAL\_INITIATION(GO:0006413)[GSEA]


|  || Dataset | readcount.sample |
| Phenotype | sample.cls |
| Upregulated in class | TE1 |
| GeneSet | TRANSLATIONAL\_INITIATION(GO:0006413) |
| Enrichment Score (ES) | 0.49116924 |
| Normalized Enrichment Score (NES) | 1.8151832 |
| Nominal p-value | 0.0 |
| FDR q-value | 0.19996014 |
| FWER p-Value | 0.494 |
Table: GSEA Results Summary

  

Fig 1: Enrichment plot: TRANSLATIONAL\_INITIATION(GO:0006413)
  
Profile of the Running ES Score & Positions of GeneSet Members on the Rank Ordered List

  

| PROBE | DESCRIPTION   (from dataset) | GENE SYMBOL | GENE\_TITLE | RANK IN GENE LIST | RANK METRIC SCORE | RUNNING ES | CORE ENRICHMENT || 1 | 103241783 | LOC103241783 |  |  | 839 | 4.783 | 0.1103 | Yes |
| 2 | 103225452 | LOC103225452 |  |  | 1425 | 2.543 | 0.1627 | Yes |
| 3 | 103243447 | EIF1 |  |  | 1639 | 2.330 | 0.2252 | Yes |
| 4 | 103231689 | EIF1AX |  |  | 1936 | 2.121 | 0.2775 | Yes |
| 5 | 103238755 | LOC103238755 |  |  | 2002 | 2.083 | 0.3389 | Yes |
| 6 | 103248689 | EIF2S2 |  |  | 2110 | 2.015 | 0.3964 | Yes |
| 7 | 103239314 | DENR |  |  | 3003 | 1.705 | 0.4092 | Yes |
| 8 | 103236007 | EIF4E |  |  | 3961 | 1.532 | 0.4138 | Yes |
| 9 | 119621207 | LOC119621207 |  |  | 4336 | 1.480 | 0.4428 | Yes |
| 10 | 103227433 | EIF1B |  |  | 5017 | 1.399 | 0.4557 | Yes |
| 11 | 103230378 | LOC103230378 |  |  | 5448 | 1.355 | 0.4783 | Yes |
| 12 | 103216053 | EIF4EBP2 |  |  | 6058 | 1.297 | 0.4912 | Yes |
| 13 | 103218088 | EIF4E2 |  |  | 6975 | 1.223 | 0.4880 | No |
| 14 | 103244656 | LOC103244656 |  |  | 9364 | 1.062 | 0.4141 | No |
| 15 | 103229755 | EIF5 |  |  | 10316 | 1.003 | 0.4026 | No |
| 16 | 103227893 | EIF4E3 |  |  | 12146 | 0.885 | 0.3483 | No |
| 17 | 103230196 | EIF2D |  |  | 13583 | 0.796 | 0.3087 | No |
| 18 | 103224961 | EIF1AD |  |  | 13813 | 0.780 | 0.3226 | No |
| 19 | 103249057 | MTIF3 |  |  | 13890 | 0.776 | 0.3431 | No |
| 20 | 103214900 | LOC103214900 |  |  | 17111 | 0.564 | 0.2167 | No |
| 21 | 103223613 | LOC103223613 |  |  | 19639 | 0.322 | 0.1137 | No |
| 22 | 103246861 | LOC103246861 |  |  | 19731 | 0.311 | 0.1193 | No |
| 23 | 103225908 | LOC103225908 |  |  | 21341 | 0.000 | 0.0474 | No |
Table: GSEA details
[plain text format]

  

Fig 2: TRANSLATIONAL\_INITIATION(GO:0006413)
  
Blue-Pink O' Gram in the Space of the Analyzed GeneSet

  

Fig 3: TRANSLATIONAL\_INITIATION(GO:0006413): Random ES distribution
  
Gene set null distribution of ES for
 **TRANSLATIONAL\_INITIATION(GO:0006413)**
